# Neuromedin B Regulates Cell Proliferation, Apoptosis and Steroidogenesis of Porcine Ovarian Granulosa Cells via NMBR

**DOI:** 10.3390/vetsci13101088

**Published:** 2026-10-14

**Authors:** Zhiyu Ma, Hongxia Li, Guangzhen Chen, Jia Li, Dandan Wang, Yiwen Wang, Sheng Yang, Qi Liu, Fenglei Chen, Shifeng Pan

**Affiliations:** 1 College of Veterinary Medicine, Yangzhou University, Yangzhou 225009, China; 2 Jiangsu Co-Innovation Center for Prevention and Control of Important Animal Infectious Diseases and Zoonoses, Yangzhou 225009, China; 3 Joint International Research Laboratory of Agriculture and Agri-Product Safety of the Ministry of Education of China, Yangzhou University, Yangzhou 225009, China

**Keywords:** NMB, NMBR, proliferation, apoptosis, steroidogenesis, granulosa cells (GCs), porcine

## Abstract

Granulosa cells (GCs) critically govern follicular development through the coordinated regulation of proliferation, apoptosis, and steroidogenesis. Neuromedin B (NMB), a local regulatory peptide, has been implicated in female reproductive processes. However, its precise effects on porcine GCs remain to be elucidated. In this study, we characterized the ovarian distribution of NMBR, investigated the effects of NMB on GC function, and further explored the involvement of NMBR in these effects using the NMBR antagonist PD168368. The results showed that higher NMBR expression was observed in follicles of larger diameter. NMB treatment differentially regulated the functional activities of porcine ovarian GCs. Specifically, certain concentrations of NMB significantly increased cell viability and altered the expression levels of the proliferation-related markers Cyclin B1 and PCNA; reduced the cell apoptosis rate and modulated the expression levels of apoptosis-related genes and proteins (Bax, Bcl-2, and Caspase-3); and affected steroidogenic responses, including increased E_2_ concentrations at selected concentrations while altering expression of STAR, CYP11A1, HSD3B1, and CYP19A1. Importantly, several of these effects were attenuated by PD168368 treatment. Collectively, these findings suggest that NMB may regulate multiple functions of porcine with the involvement of NMBR. These findings provide a theoretical basis for elucidating the neuroendocrine mechanisms underlying porcine follicular development and for further investigating the potential role of NMB/NMBR signaling in porcine follicular physiology.

## Simple Summary

Neuromedin B is a naturally occurring signaling molecule that may be involved in the regulation of ovarian function, but its role in the pig ovary is not well understood. This study examined where the receptor for neuromedin B is located in the pig ovary and investigated how neuromedin B affects granulosa cells, which are cells surrounding the developing egg and are important for follicle growth and hormone production. We evaluated its effects on cell growth, cell death, and the production of steroid hormones, which are essential for normal reproductive function. We also used a compound that blocks the neuromedin B receptor to determine whether this receptor is involved in these effects. The results showed that an appropriate concentration of neuromedin B promoted granulosa cell growth, reduced cell death, and increased steroid hormone production, whereas blocking its receptor partly weakened these effects. These findings improve our understanding of how ovarian function is regulated in pigs and may provide useful information for future studies aimed at improving reproductive health and efficiency in pigs.

## 1. Introduction

Ovarian follicles are the basic functional units of the ovary, and their cyclic development and atresia govern ovarian endocrine function and directly influence the reproductive performance of female animals [1]. Granulosa cells (GCs), the major somatic cell population within ovarian follicles, surround the oocyte and play a crucial role in supporting oocyte development, follicular cavity formation, and the maintenance of follicular microenvironment homeostasis [2,3]. The GC proliferation, apoptosis, and steroidogenesis are closely associated with follicular developmental status. During the transition from primordial follicles to preovulatory follicles, GCs exhibit significant proliferative and differentiative capabilities. Cell proliferation is typically characterized by the initiation of DNA replication, cell-cycle progression and an increase in cell number. Among these, proliferating cell nuclear antigen (PCNA) serves as a key cofactor in DNA replication, while Cyclin B1 is involved in the G2/M transition [4,5]. Accordingly, the expression levels of PCNA and Cyclin B1 are commonly used to assess the proliferative status of GCs [6]. In contrast, GCs apoptosis is the primary cause of follicular atresia, and its occurrence is jointly regulated by the mitochondrial pathway and the caspase cascade [7]. The Bax/Bcl-2 ratio is widely used as an indicator of apoptotic susceptibility, whereas Caspase-3, a key executioner caspase, is closely associated with GCs apoptosis [8,9,10,11]. GCs are also essential functional cells involved in porcine ovarian steroidogenesis. This process is regulated by several key steroidogenic proteins, including steroidogenic acute regulatory protein (STAR), cytochrome P450 family 11 subfamily A member 1 (CYP11A1), hydroxy-delta-5-steroid dehydrogenase, 3 beta- and steroid delta-isomerase 1 (HSD3B1) and cytochrome P450 family 19 subfamily A member 1 (CYP19A1) [12]. These factors coordinately contribute to the biosynthesis of progesterone (P_4_) and estradiol (E_2_). Impaired aromatase expression or activity can disrupt E_2_ biosynthesis and may consequently contribute to reproductive dysfunction [13,14]. Furthermore, the local ovarian microenvironment contains a variety of autocrine and paracrine factors that collectively regulate GCs function [15,16]. Therefore, further elucidation of the mechanisms through which these local regulatory factors modulate GCs proliferation, apoptosis, and steroidogenesis is important for advancing our understanding of the molecular regulatory networks underlying porcine follicular development and atresia.

Neuromedin B (NMB), a member of the mammalian bombesin-related peptide family, exerts its biological effects by binding to its neuromedin B receptor (NMBR). NMBR is a G protein-coupled receptor that, upon ligand binding, activates intracellular signaling pathways involved in calcium mobilization, cell proliferation, energy metabolism, and endocrine regulation. NMB acts as a local autocrine and paracrine factor that regulates a variety of cellular functions. For example, in the rat pituitary, NMB has been reported to act as an autocrine/paracrine inhibitor of thyrotropin (TSH) secretion [17]. NMB derived from cervical cancer cells activates NMBR in Schwann cells and promotes the opening of T-type Ca^2+^ channels via protein kinase A (PKA) signaling, thereby stimulating Ca^2+^ influx [18]. Additionally, this effect can be blocked by pharmacological inhibition. Previous studies have implicated the NMB/NMBR system in a broad range of physiological processes, including neuromodulation, gastrointestinal function, metabolic regulation, smooth muscle contraction, and endocrine activity [19,20,21,22]. In recent years, the potential role of the NMB/NMBR system in reproductive regulation has gradually attracted attention. NMB and NMBR are expressed in components of the hypothalamic–pituitary–gonadal axis in mammals, and their expression in the porcine reproductive axis displays developmental dynamics [22,23]. NMB can influence the proliferation, apoptosis, testosterone synthesis and secretion of Leydig cells in porcine testes [24]. However, current research on NMB in female reproductive regulation is relatively limited, particularly in large livestock, and particularly in pigs. In our separate study, NMB expression was found to increase with increasing follicular diameter in pigs. However, the expression pattern of its receptor, NMBR, during porcine follicular development remains unclear. Whether the NMB/NMBR system directly regulates follicular development and GCs function in pigs has not yet been systematically elucidated. Notably, recent evidence in goats indicates that NMB/NMBR signaling can directly modulate GCs function. Exogenous NMB promotes GCs proliferation, increases the proportion of cells in S phase, and upregulates the expression of cell-cycle-related molecules, whereas NMBR antagonism attenuates these effects [25]. However, whether similar regulatory effects occur in the porcine ovary, particularly with respect to the coordinated regulation of GCs proliferation, apoptosis, steroidogenesis, and steroid hormone secretion, remains to be determined.

Building on these observations, this study first investigated the distribution of NMBR in porcine ovarian tissue and compared the gene and protein expression levels of NMBR in follicles of different diameters. Building on these findings, an in vitro culture model of porcine ovarian GCs was established to systematically evaluate the effects of NMB on cell proliferation, apoptosis, and steroidogenesis. Additionally, co-treatment with NMB and the NMBR antagonist was used to determine whether the observed effects of NMB were mediated by NMBR. This study aimed to advance the understanding of the molecular mechanisms through which NMB regulates porcine ovarian GCs function. It may also provide insight into the local regulatory role of NMB/NMBR signaling in porcine follicular development and offer a foundation for elucidating the mechanisms underlying follicular development and atresia, as well as for identifying potential targets for reproductive regulation.

## 2. Materials and Methods

### 2.1. Materials

Porcine NMB-10 (purity ≥ 98% by HPLC analysis, 1240513, Shanghai Jier Bio-Chem Co., Ltd., Shanghai, China), the NMBR receptor antagonist PD168368 (HY-116216, MCE, Shanghai, China), dimethyl sulfoxide (DMSO, ST038-100 mL, Beyotime Biotechnology, Shanghai, China), penicillin–streptomycin solution (PS, ST488S, Beyotime Biotechnology), SABC immunohistochemical staining kit (SA1020, BOSTER, Wuhan, China), DAB (AR1022, BOSTER), DMEM/F-12 (1:1) medium (C11330500BT, Gibco, Suzhou, China), fetal bovine serum (FBS, EallBio, Beijing, China), RNAiso Plus (AJ60718A, TaKaRa, Beijing, China), PrimeScript FAST RT reagent Kit with gDNA Eraser (RR092S, TaKaRa), ChamQ Blue Universal SYBR qPCR Master Mix (Q312, Vazyme, Nanjing, China), BCA Protein Concentration Assay Kit (P0010, Beyotime Biotechnology), ECL Ultra-Sensitive Chemiluminescent Reagent (P10300, NCM Biotech, Suzhou, China), Enhanced CCK-8 Kit (C0042, Beyotime Biotechnology), the Annexin V-FITC Apoptosis Detection Kit (C1062L, Beyotime Biotechnology), BeyoPure™ Ultrapure Water (Suitable for Cell Culture) (ST875, Beyotime Biotechnology), Protease and Phosphatase Inhibitor Cocktail (P002, NCM Biotech), RIPA lysis buffer (P0013B, Beyotime Biotechnology).

### 2.2. Sample Collection

The porcine ovarian tissue was provided by Yangzhou Xiangtai Food Co., Ltd. (Yangzhou, China) and was sourced from pigs of the same breed and age as the whole-body tissue samples, thereby ensuring comparability among the samples. The use of these samples did not involve any additional animal experiments or interventions, nor did it involve experiments on live animals. The collected porcine ovaries were placed in 37 °C saline solution and transported back to the laboratory within one hour. Follicles were carefully dissected from the pig ovaries using pointed and curved forceps. Healthy follicles were selected under a stereomicroscope (intact follicular wall, clear vascularization, transparent follicular fluid, and no hemorrhage or turbidity) [26]. Follicles showing obvious signs of atresia were excluded. The follicular diameters were measured and classified into three size categories (<3 mm, 3–5 mm, >5 mm) [27]. The isolated follicles were placed in EP tubes, labeled, rapidly frozen in liquid nitrogen, and stored at −80 °C for subsequent genetic and protein analysis. The porcine ovarian tissue was promptly placed in an appropriate fixative (4% paraformaldehyde) for immunohistochemical analysis.

### 2.3. Cell Culture and Treating

The mesovarium and attached oviductal tissues were removed from porcine ovaries. The ovaries were placed in sterile saline supplemented with 1% PS and prewarmed to approximately 38 °C. They were gently rinsed two to three times to remove residual blood until the washing solution became clear and were then transported to the cell culture laboratory. Subsequently, the ovaries were immersed in 75% ethanol for 10 s, immediately transferred to sterile saline, and rinsed twice. The ovaries were then maintained in a 38 °C water bath until further processing. Healthy follicles with diameters of 3–5 mm were selected, and follicular fluid was aspirated using a 5 mL syringe and collected in 15 mL centrifuge tubes. The samples were centrifuged at 1500 *g* for 5 min, after which the supernatant was discarded and the cell pellet was collected. The cell pellet was resuspended in DMEM/F12 medium, and the cell suspension was filtered through a 40 μm cell strainer. The filtrate was centrifuged, and the resulting cell pellet, containing porcine GCs, was collected. For each biological replicate, ovaries obtained from multiple pigs were pooled for GC isolation, and each replicate was prepared independently. Three independent pooled biological replicates were used in the experiments (n = 3 biological replicates). The cells were resuspended in complete DMEM/F12 medium supplemented with 10% FBS and 1% PS. Viable cells were counted and seeded uniformly into culture plates at a density of 1 × 10^6^ cells/mL. The cells were cultured at 37 °C in a humidified atmosphere containing 5% CO_2_. Cell morphology and growth were monitored daily, and the culture medium was replaced with fresh complete medium as required. After seeding, the cells were allowed to attach and recover for at least 24 h. Depending on cell attachment and confluence, experimental treatments were initiated approximately 24–36 h after seeding, when the cells had reached 70–80% confluence. The cells were maintained and treated in complete DMEM/F12 medium containing 10% FBS and 1% PS throughout the experiments. Within each independent experiment, the control and treatment groups were cultured under the same serum-containing conditions to maintain a consistent culture background.

PD168368 was dissolved in DMSO to a concentration of 5 mmol/L to prepare a stock solution, which was then diluted in cell culture medium to 1000 nmol/L. This solution was added to cells after fresh culture medium had been replaced, and the cells were pretreated for 1 h before NMB treatment. Cells were treated with porcine NMB-10; the NMB peptide was diluted with BeyoPure™ Ultrapure Water (Suitable for Cell Culture) to prepare a stock solution of NMB peptide at a concentration of 5 × 10^5^ nmol/L. The NMB peptide was then diluted in cell culture medium to concentrations of 0 nmol/L, 0.1 nmol/L, 1 nmol/L, 10 nmol/L, 100 nmol/L, and 1000 nmol/L, and added to the corresponding cell wells. The NMB concentration range (0.1–1000 nmol/L) was selected based on preliminary experiments conducted in our laboratory to evaluate cellular responses over a broad concentration range. The cells were incubated in the incubator for 24 h before further analysis.

### 2.4. Quantitative Real-Time PCR (qPCR)

Total RNA was extracted according to the manufacturer’s instructions for the RNA extraction kit. RNA concentration and purity were determined using a UV spectrophotometer, and RNA integrity was assessed by agarose gel electrophoresis. Subsequently, total RNA was reverse-transcribed into complementary DNA (cDNA) using the PrimeScrip^TM^ FAST RT Reagent Kit with gDNA Eraser. The synthesized cDNA was stored at −20 °C until further analysis. Quantitative real-time PCR (qPCR) was performed using ChamQ Blue Universal SYBR qPCR Master Mix according to the manufacturer’s instructions. Complementary DNA (cDNA) synthesized by reverse transcription was used as the template, and the primer sequences are listed in Table 1. The primer stock solutions were prepared at 100 μM and diluted to 10 μM working solutions before use. The final concentration of each primer in the reaction was 0.5 μM. Each qPCR reaction was conducted in a final volume of 10 μL containing 5.0 μL of 2× ChamQ Blue Universal SYBR qPCR Master Mix, 0.5 μL of 10 μM forward primer, 0.5 μL of 10 μM reverse primer, 1.0 μL of cDNA template, and 3.0 μL of nuclease-free water. The amplification protocol consisted of an initial denaturation step at 95 °C for 5 min, followed by 40 cycles of denaturation at 95 °C for 10 s and annealing/extension at 60 °C for 30 s. Melting-curve analysis was subsequently performed at 95 °C for 15 s, 60 °C for 30 s, and 95 °C for 10 s to verify amplification specificity. *β-actin* was used as the internal reference gene. Each sample was analyzed in triplicate. Relative mRNA expression levels of the target genes were calculated using the 2^−^^ΔΔCt^ method.

### 2.5. Western Blot Assay

Protease and phosphatase inhibitor cocktails were added to RIPA lysis buffer prior to use. The samples were lysed on ice for 10 min and centrifuged at 12,000× *g* at 4 °C, after which the supernatants were collected. Protein concentrations were determined using a BCA protein assay kit according to the manufacturer’s instructions. Briefly, an aliquot of each protein lysate was used for protein quantification, and the total protein concentration was calculated from a protein standard curve. The protein samples were mixed with 5 × SDS-PAGE loading buffer, denatured by boiling, and stored at −80 °C until further use.

A 10% polyacrylamide gel was prepared using the Omni-Easy™ One-Step PAGE Gel Rapid Preparation Kit according to the manufacturer’s instructions. Equal amounts of total protein (20 μg per lane) were separated by electrophoresis on the 10% PAGE gel at 80 V. Once the samples had migrated into the resolving gel, the voltage was increased to 120 V. After electrophoresis, the gel was cut into separate regions according to the molecular-weight positions indicated by the prestained protein marker and the expected molecular weights of the target proteins and β-actin. The proteins were then transferred onto polyvinylidene difluoride (PVDF) membranes, which had been pre-activated in methanol for 3 min. The membranes were blocked with 5% skim milk for 2 h and washed three times with Tris-buffered saline containing 0.1% Tween-20 (TBST) for 10 min each. Subsequently, the PVDF membranes were incubated overnight at 4 °C with appropriately diluted primary antibodies. After washing with TBST to remove unbound primary antibodies, the membranes were incubated with the corresponding secondary antibodies at room temperature for 45 min. The membranes were then washed with TBST to remove unbound secondary antibodies. An appropriate volume of enhanced chemiluminescence (ECL) substrate was evenly applied to the PVDF membranes. Specific protein signals were detected using a fully automated chemiluminescence imaging system, and the images were saved for subsequent analysis. Band intensities were quantified using ImageJ 1.54g. The expression level of each target protein was normalized to the corresponding β-actin signal from the same sample, and the normalized values were then expressed relative to the control group. The specificity of the primary antibodies was assessed based on the validation information provided by the manufacturers and whether the detected immunoreactive bands corresponded to the expected molecular weights of the target proteins. Detailed information on the antibodies used in this study is provided in Table 2.

### 2.6. Immunohistochemistry (IHC)

Following the protocol described previously by Ma et al. [22], porcine ovarian tissues were fixed in 4% paraformaldehyde, embedded in paraffin, and sectioned. IHC staining was performed using the SABC IHC Kit according to the manufacturer’s instructions. Paraffin sections were first incubated at 60 °C for 1 h, deparaffinized in xylene, and rehydrated through a graded ethanol series. Endogenous peroxidase activity was quenched by incubation with 3% H_2_O_2_ at room temperature for 10 min. The sections were then washed three times with phosphate-buffered saline (PBS) for 5 min each. For antigen retrieval, the sections were immersed in citrate buffer, heated to a gentle boil, and allowed to cool to room temperature. This procedure was repeated three times, followed by three washes with PBS for 5 min each. The sections were subsequently blocked with 5% bovine serum albumin (BSA) at 37 °C for 30 min, after which excess blocking solution was removed. The sections were incubated overnight at 4 °C with appropriately diluted primary antibodies. For the negative controls, PBS was substituted for the primary antibody. After washing three times with PBS for 5 min each, the sections were incubated with a biotinylated secondary antibody according to the manufacturer’s instructions and washed with PBS. The sections were then developed with 3,3′-diaminobenzidine (DAB) substrate solution. The chromogenic reaction was terminated by rinsing with tap water, and the sections were counterstained with hematoxylin, dehydrated through a graded ethanol series, cleared in xylene, and mounted. Images were acquired using an upright light microscope and saved for subsequent analysis. Detailed information on the antibodies used in this study is provided in Table 2.

### 2.7. Immunocytochemistry (ICC)

To assess the identity of the cultured porcine ovarian granulosa cells (GCs), FSHR immunocytochemical staining was performed. Porcine ovarian GCs suspensions were seeded into 24-well plates containing sterile glass coverslips and cultured for 24 h until the cells reached approximately 50–60% confluence. immunocytochemical staining was subsequently performed using the SABC IHC kit according to the manufacturer’s instructions.

Briefly, the cells were fixed with 4% paraformaldehyde for 20 min, and endogenous peroxidase activity was blocked with 3% H_2_O_2_. The cells were then permeabilized with 0.25% Triton X-100 and blocked with 5% BSA. Thereafter, the cells were sequentially incubated with the primary antibody against FSHR and secondary antibodies, developed with DAB, mounted, and imaged. Detailed procedures were performed as described in Section 2.6. FSHR-positive staining, together with the characteristic morphology of the cultured cells, was used to support the granulosa-cell identity of the isolated cells.

### 2.8. Cell Viability Assay

Porcine ovarian GCs were uniformly seeded into 96-well plates at a volume of 100 μL per well. The peripheral wells were filled with sterile PBS to minimize evaporation. The cells were cultured until they reached approximately 70–80% confluence. The cells were then treated with PD168368 and NMB and incubated at 37 °C in a humidified atmosphere containing 5% CO_2_ for 24 h. Subsequently, 10 μL of CCK-8 solution was added to each well. The plates were gently shaken to ensure complete mixing while avoiding bubble formation and were incubated in the dark for 1–4 h. Absorbance was measured at 450 nm using a microplate reader.

### 2.9. Cell Apoptosis Assay

Porcine ovarian GCs apoptosis was assessed using an Annexin V-FITC Apoptosis Detection Kit according to the manufacturer’s instructions. Briefly, pre-chilled PBS was used to wash the cells twice. The cells were then digested with EDTA-free trypsin for 7–8 min, and the digestion was terminated by the addition of serum-containing culture medium. The cell suspension was collected into 1.5 mL centrifuge tubes and centrifuged at 1500× *g* for 3 min. The resulting cell pellets were gently resuspended in 195 μL of Annexin V-FITC binding buffer, followed by the addition of 5 μL of Annexin V-FITC and 10 μL of propidium iodide (PI) staining solution. The samples were gently mixed and incubated at room temperature in the dark for 10 min. Apoptotic cells were subsequently analyzed by flow cytometry, and the data were processed using FlowJo 10.8.1 software. In the flow cytometric analysis, Q3 represented early apoptotic cells, Q2 represented late apoptotic cells, Q1 represented mechanically damaged or necrotic cells, and Q4 represented viable cells. The total apoptotic cell percentage was calculated as Q2 + Q3. For relative comparison among treatment groups, the ratio of apoptotic cells to viable cells was further calculated as (Q2 + Q3)/Q4. This value was then normalized to the control group, which was set to 100%. The relative apoptotic level was calculated as follows: Relative apoptotic level (%) = [(Q2 + Q3)/Q4] treatment/[(Q2 + Q3)/Q4] control × 100.

### 2.10. Steroid Hormone Testing

Cell culture supernatants were collected from each group, transferred to centrifuge tubes, and centrifuged at 4 °C to remove cellular debris and suspended impurities. The clarified supernatants were retained for subsequent analysis. The concentrations of E_2_ and P_4_ in the cell culture supernatants were determined by Yangzhou Hengyi Biotechnology Co., Ltd. (Yangzhou, China). The linear detection ranges were 20.0–2000.0 pg/mL for E_2_ and 0.35–65.00 ng/mL for P_4_. The linear correlation coefficients for both assays were not less than 0.9900. The coefficients of variation (CVs) for repeatability and inter-batch variation were ≤8.0% and ≤15.0%, respectively, and the relative deviations were within ±15.0%.

### 2.11. Statistical Analysis

Image processing and analysis were performed using Adobe Photoshop 2020 and ImageJ. Data are presented as the mean ± standard error of the mean (SEM) from three independent biological replicates (n = 3). Graphs were generated using GraphPad Prism 5.0, and significance tests for differences between groups were conducted using one-way ANOVA in SPSS Statistics 17.0. Differences between two groups were analyzed using an independent-samples t-test. For comparisons among multiple groups, one-way analysis of variance (ANOVA) was performed, followed by Tukey’s post hoc test for pairwise comparisons. A *p* value < 0.05 was considered statistically significant, and *p* < 0.01 was considered highly significant. In the figures, * indicates *p* < 0.05 and ** indicates *p* < 0.01. For the NMBR antagonist experiments, different superscript letters indicate significant differences between groups based on Tukey’s multiple-comparison test (*p* < 0.05), whereas groups sharing the same letter are not significantly different.

## 3. Results

### 3.1. Expression Pattern of NMBR in Porcine Follicles of Different Diameters

**Figure 1:**
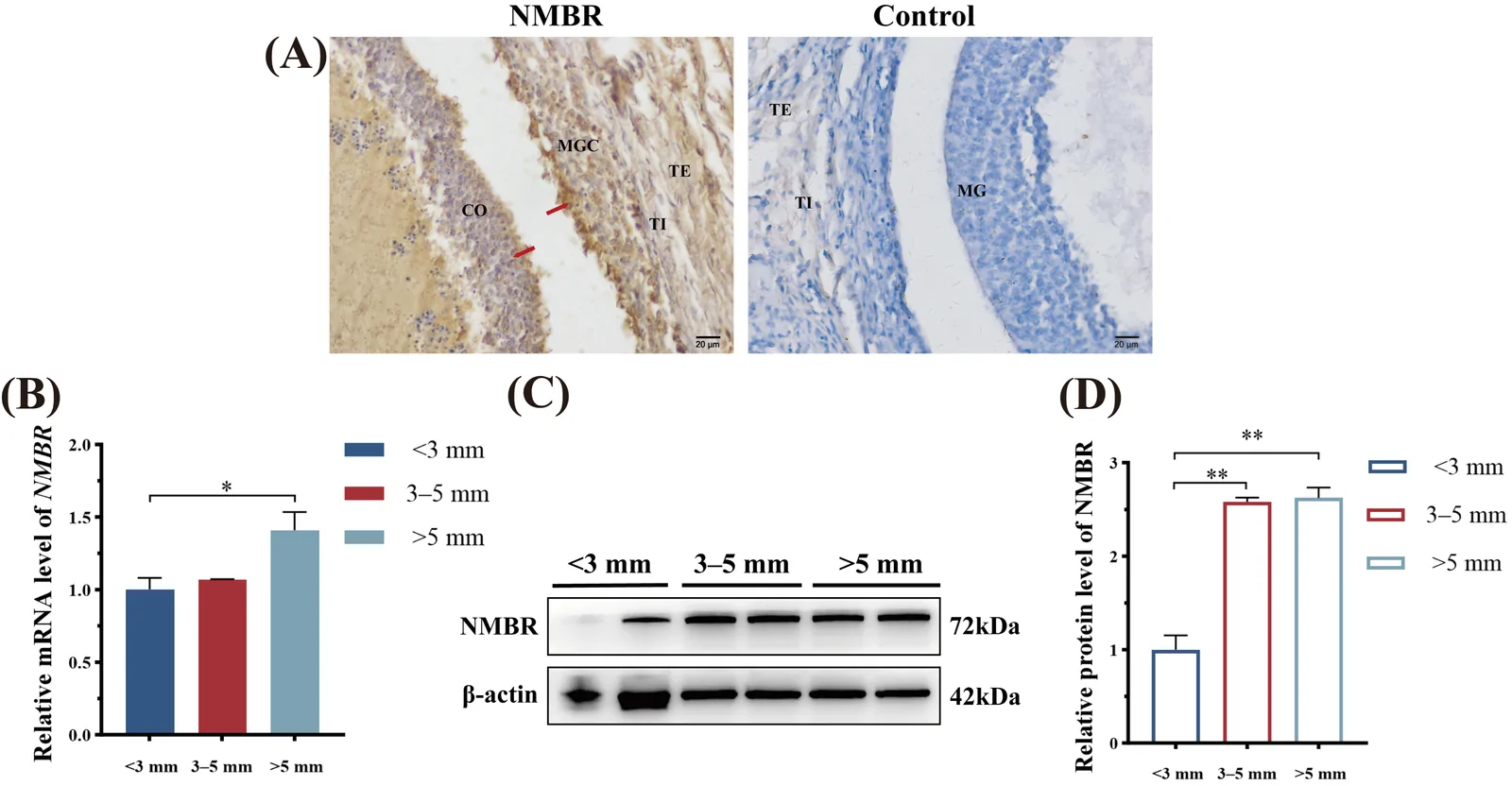
The expression level of NMBR in porcine follicles. (**A**) Representative NMBR immunohistochemical staining and the corresponding negative control without primary antibody. (**B**) The mRNA expression levels of the *NMBR* gene in porcine follicles in different diameters. (**C**,**D**) The protein expression levels of NMBR in porcine follicles in different diameters. CO: cumulus oophorus, MGC: medial granulosa cells, TI: inner theca, TE: outer theca, MG: membrana granulosa; the scale bar in Figure 1A is 20 μm. Data are presented as the mean ± SEM from three independent biological replicates (n = 3), with each sample measured in duplicate within each independent experiment. Compared to the expression levels in <3 mm follicle group, * *p* < 0.05, ** *p* < 0.01. Original uncropped Western blot images corresponding to Figure 1C are provided in Supplementary Figure S1.

Analysis of NMB and NMBR protein expression in various porcine tissues revealed that both proteins were highly expressed in the ovaries. To identify the ovarian cell types potentially targeted by NMB and its receptor basis, this study used IHC to examine the localization and distribution of NMBR in porcine ovarian tissue. IHC staining showed that NMBR was expressed in porcine ovarian follicles, with positive staining observed in GCs and the surrounding theca layers (Figure 1A). Accordingly, this study further isolated follicles of different diameters from porcine ovaries and used qPCR and Western blot methods to detect *NMBR* gene and protein expression levels in follicles of different diameters, thereby investigating its expression patterns across follicles of varying sizes. The results of *NMBR* gene analysis showed that, compared with follicles <3 mm, *NMBR* gene expression levels in follicles > 5 mm exhibited significant differences (*p* < 0.05), while no significant differences were observed in follicles 3–5 mm in diameter (Figure 1B). The results of NMBR protein detection showed that the NMBR protein expression levels in 3–5 mm and >5 mm follicles differed significantly from those in <3 mm follicles (*p* < 0.01; Figure 1C,D). Original uncropped Western blot images corresponding to Figure 1C are provided in Supplementary Figure S1. These results indicate that NMBR expression levels tend to increase with increasing follicle diameter.

### 3.2. Establishment and Characterization of Primary Porcine GCs

The results described above showed that NMBR was clearly expressed in porcine ovarian tissue. Moreover, Higher NMBR expression was observed in follicles of larger diameter, suggesting that the NMB/NMBR signaling system may be involved in porcine follicular development. Although NMBR was also detectable in the theca compartment, the present study focused on granulosa cells because of their important role in follicular growth, survival, and steroidogenesis. However, whether NMB can directly act on porcine GCs and regulate their biological functions remains unclear. Therefore, primary porcine GCs were further isolated and cultured in vitro. The ICC staining of FSHR was used to identify the cultured cells, and NMBR expression and localization were examined to provide an experimental basis for subsequent NMB stimulation assays.

**Figure 2:**
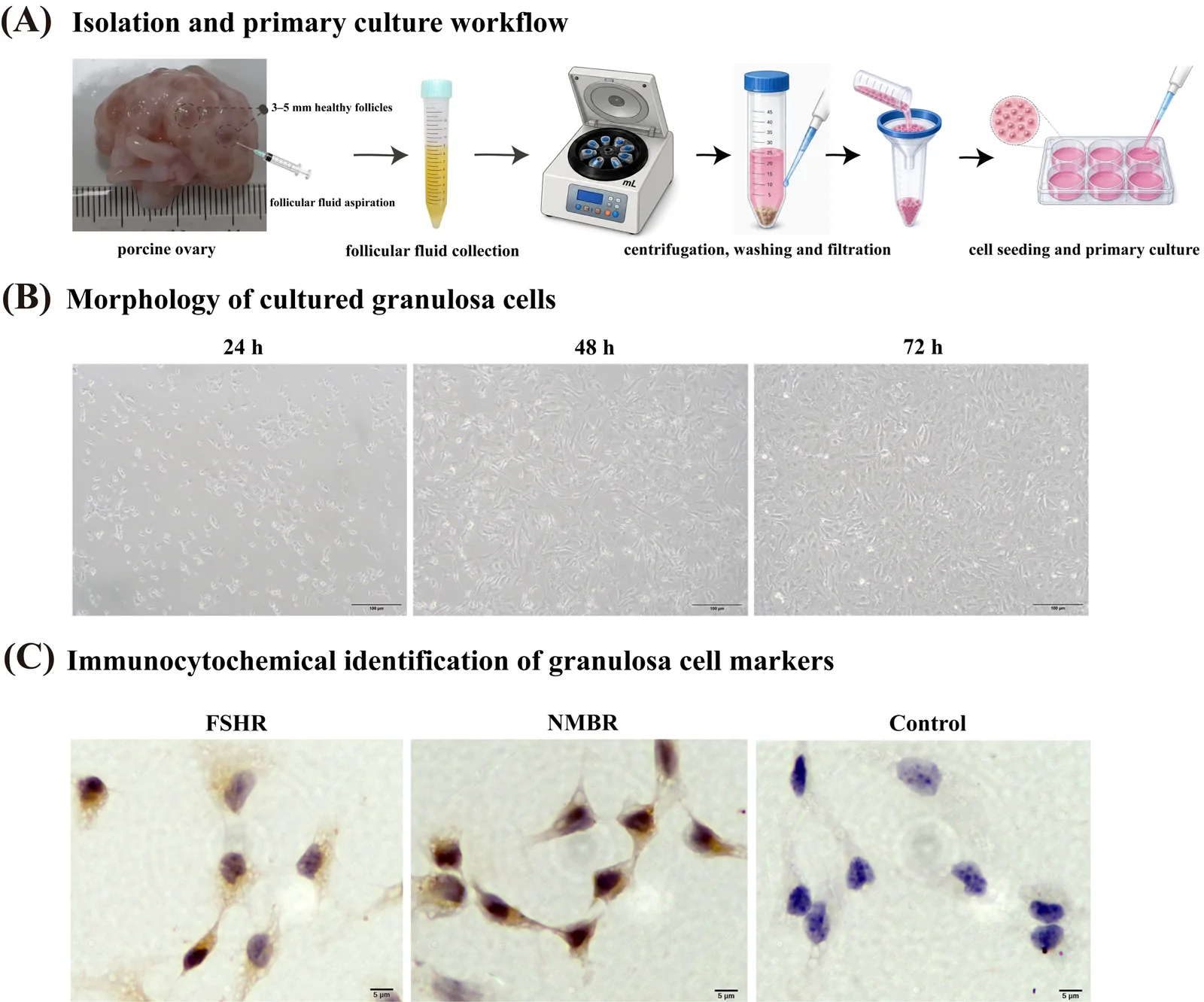
Establishment of a primary porcine ovarian GC model. (**A**) Schematic workflow showing follicular fluid collection, centrifugation, washing, filtration, and primary culture of porcine ovarian GCs. (**B**) Morphology of porcine ovarian GCs. Scale bar = 100 μm. (**C**) Immunocytochemical image of NMBR and FSHR proteins. Negative control staining was included to assess nonspecific background staining. Scale bar = 5 μm.

Primary porcine ovarian GCs were isolated by aspirating follicular fluid from healthy 3–5 mm follicles using a sterile syringe, followed by in vitro culture of the collected cells (Figure 2A). Microscopic observation showed that the cultured cells grew as an adherent monolayer, with relatively large cell bodies, intact morphology, and clearly defined cell borders (Figure 2B). Most cells exhibited spindle-like, stellate, polygonal, or triangular morphology. Primary porcine GCs showed progressive attachment, spreading, and proliferation from 24 to 72 h after seeding, forming a nearly confluent monolayer by 72 h.

ICC staining was further performed to assess granulosa cell identity and to determine the expression and distribution of NMBR protein in the cultured cells (Figure 2C). Obvious FSHR-positive staining was detected in the cultured porcine ovarian granulosa cells, with brown DAB signals mainly distributed in the cytoplasm, while the nuclei were counterstained blue-purple with hematoxylin. NMBR-positive staining was also observed, indicating that the cultured cells expressed NMBR protein. In contrast, the negative control showed only nuclear counterstaining without obvious specific brown staining, suggesting a low level of nonspecific background signal. Together, these findings support the granulosa cell phenotype of the primary cultured cells and indicate that they possess the receptor basis for responding to NMB, making them suitable for subsequent in vitro experiments.

### 3.3. NMB Regulates Cell Proliferation of Porcine GCs

**Figure 3:**
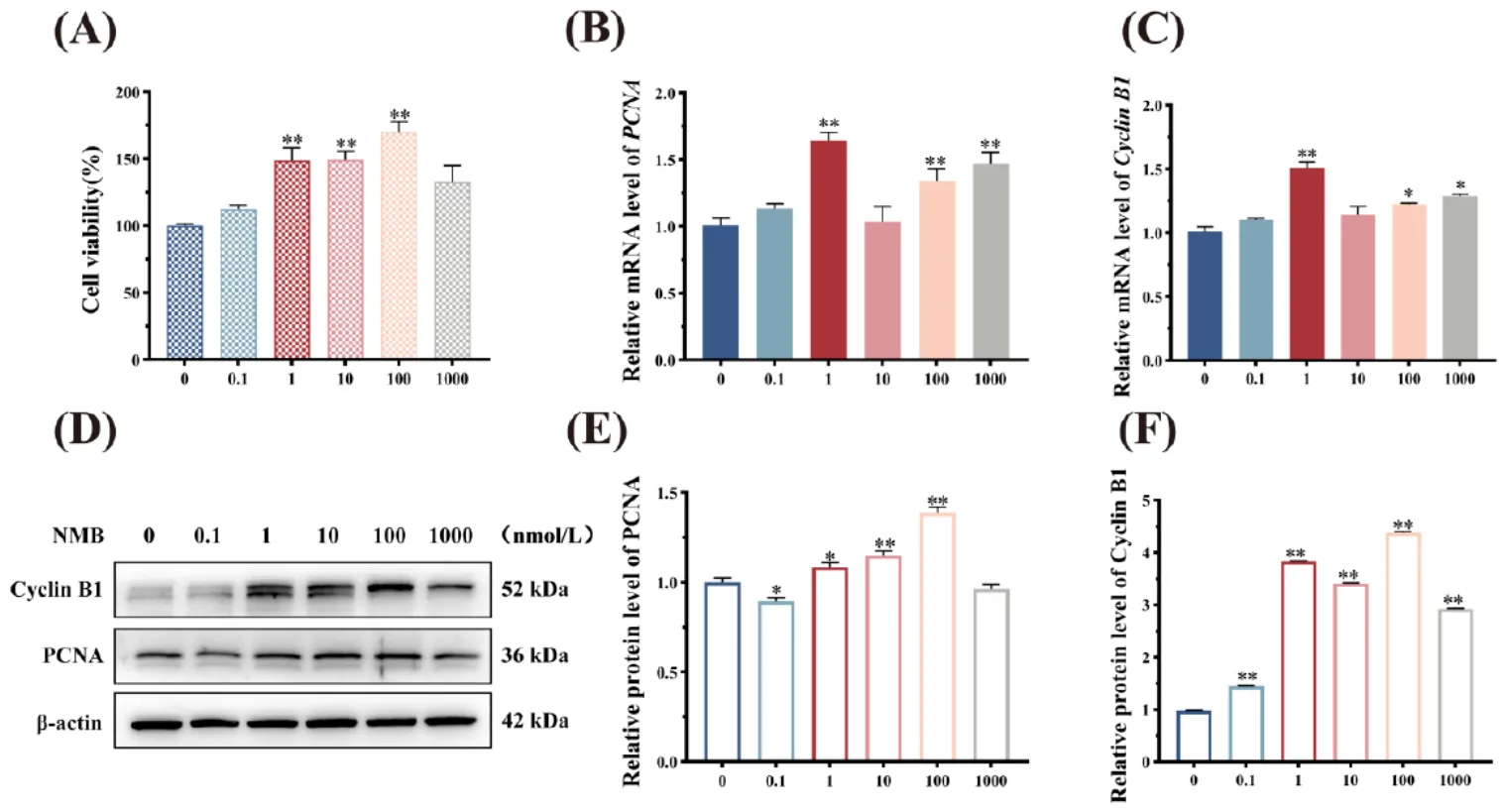
Effects of different concentrations of NMB on cell proliferation in porcine GCs. (**A**) Effects of different concentrations of NMB on cell viability of porcine GCs. (**B**) Effect of different concentrations of NMB on *PCNA* mRNA expression. (**C**) Effect of different concentrations of NMB on *Cyclin B1* mRNA expression. (**D**) Western blot detected the expression of PCNA and Cyclin B1 proteins at different concentrations of NMB. (**E**) Gray-scale analysis of PCNA protein. (**F**) Gray-scale analysis of Cyclin B1 protein. Data are presented as the mean ± SEM from three independent biological replicates (n = 3), with each sample measured in duplicate within each independent experiment. Compared with the control group, * *p* < 0.05, ** *p* < 0.01. Original uncropped Western blot images corresponding to Figure 3D are provided in Supplementary Figure S2.

To investigate the effects of NMB on porcine ovarian GCs, primary GCs were treated with different concentrations of NMB for 24 h. The CCK-8 assay was used to assess the effects of NMB on the cell viability of GCs. The results showed (Figure 3A) that NMB increased the cell viability of primary GCs to varying degrees. Specifically, compared with the control group, cell activities in the 1, 10 and 100 nmol/L NMB-treated groups was all significantly elevated (*p* < 0.01), suggesting that NMB at certain concentrations may promote cell proliferation, whereas 0.1 and 1000 nmol/L NMB had no significant effect on cell activity.

Further analysis of proliferation-related genes (*PCNA* and *Cyclin B1*) revealed that, as shown in the *PCNA* results (Figure 3B), compared with the control group, the *PCNA* gene expression was significantly increased in the 1, 100, and 1000 nmol/L NMB-treated groups (*p* < 0.01), while there was little difference between the remaining treatment groups and the control group. Compared with the control group, 1 nmol/L NMB significantly promoted the mRNA expression level of *Cyclin B1* (*p* < 0.01); 100 and 1000 nmol/L NMB significantly increased *Cyclin B1* mRNA expression levels (*p* < 0.05), while the remaining concentrations of NMB showed no significant difference in their stimulatory effects on this gene (Figure 3C). Similarly, the effect of NMB on the *Cyclin B1* gene was similar to that on *PCNA*.

PCNA protein analysis revealed that (Figure 3D,E), compared with the control group, both 10 and 100 nmol/L NMB significantly increased its expression (*p* < 0.01), and 1 nmol/L NMB also significantly increased its expression (*p* < 0.05). However, treatment with 0.1 nmol/L NMB significantly inhibited PCNA protein expression (*p* < 0.05). Compared with the control group, NMB treatment significantly promoted Cyclin B1 protein expression (Figure 3D,F) (*p* < 0.01). Original uncropped Western blot images corresponding to Figure 3D are provided in Supplementary Figure S2.

### 3.4. NMB Regulates Apoptosis of Porcine GCs

**Figure 4:**
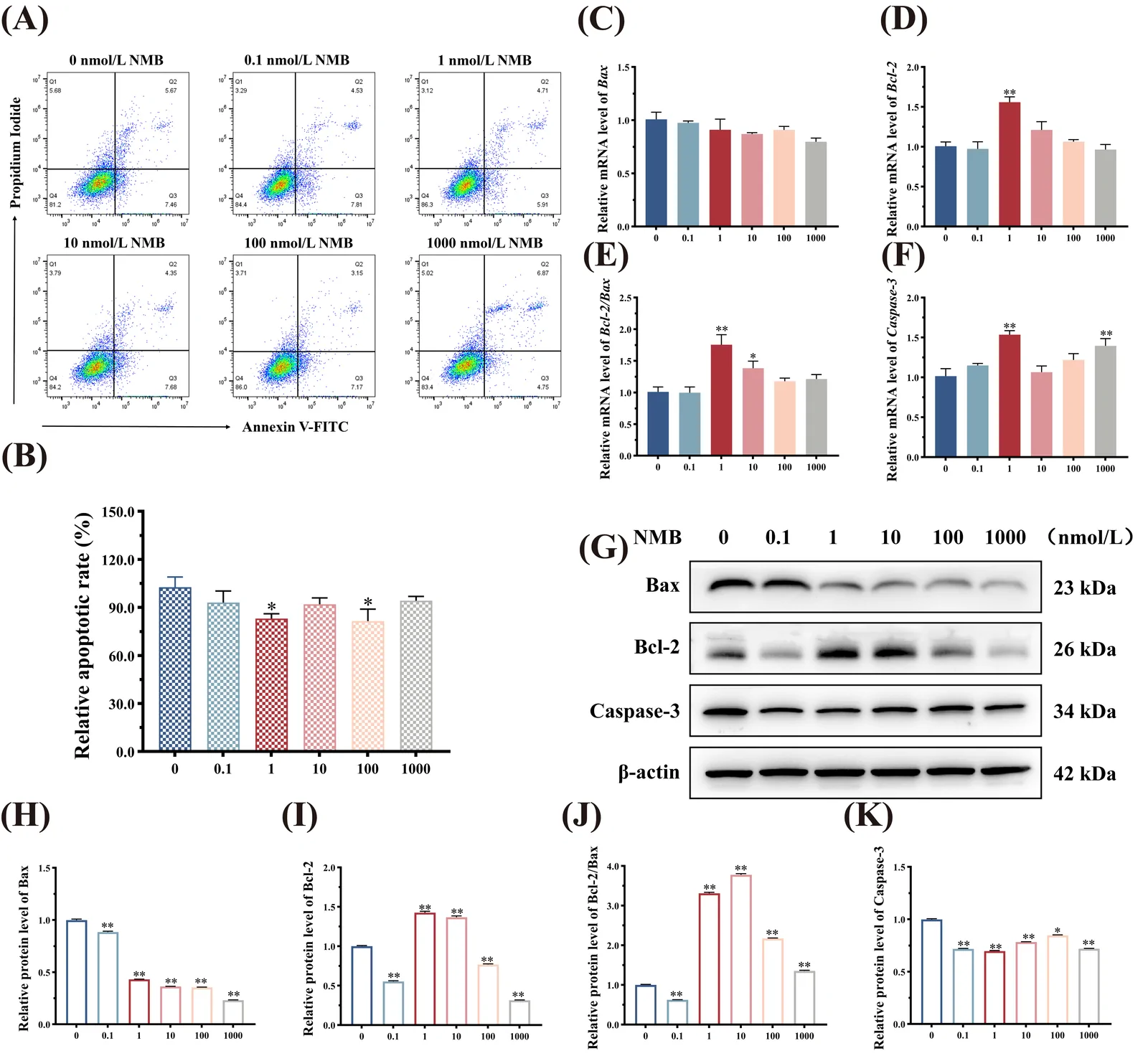
Effects of different concentrations of NMB on cell apoptosis of porcine GCs. (**A**) Representative results of flow cytometry assay. (**B**) The quantification of apoptotic cell populations. (**C**) Effect of different concentrations of NMB on *Bax* mRNA expression. (**D**) Effect of different concentrations of NMB on *Bcl-2* mRNA expression. (**E**) Effect of different concentrations of NMB on *Bcl-2/Bax* mRNA expression. (**F**) Effect of different concentrations of NMB on *Caspase-3* mRNA expression. (**G**) Western blot detected the expression of Bax, Bcl-2, and Caspase-3 proteins at different concentrations of NMB. (**H**) Gray analysis of Bax protein. (**I**) Gray analysis of Bcl-2 protein. (**J**) Gray analysis of Bcl-2/Bax protein. (**K**) Gray analysis of Caspase-3 protein. Data are presented as the mean ± SEM from three independent biological replicates (n = 3), with each sample measured in duplicate within each independent experiment. Compared with the control group, * *p* < 0.05, ** *p* < 0.01. Original uncropped Western blot images corresponding to Figure 4G are provided in Supplementary Figure S3.

Since granulosa cell apoptosis is one of the key factors affecting follicular development, this study further used flow cytometry to examine the effects of different concentrations of NMB on the apoptosis rate of porcine GCs. As shown in Figure 4A, stimulation with certain concentrations of NMB could inhibit apoptosis in porcine GCs. Compared with the control group, 1 and 100 nmol/L NMB significantly inhibited apoptosis in porcine GCs (*p* < 0.05), whereas 0.1, 10 and 1000 nmol/L had no significant effect (Figure 4B).

To further investigate the underlying molecular mechanisms by which NMB affects apoptosis in porcine GCs, this study examined the effects of NMB treatment on apoptosis-related genes (*Bax*, *Bcl-2*, and *Caspase-3*) and *Bcl-2/Bax* expression. The results showed that, compared with the control group, NMB treatment suppressed *Bax* mRNA expression levels, but the difference was not significant (*p* > 0.05) (Figure 4C). Treatment with 1 nmol/L NMB significantly increased *Bcl-2* mRNA expression levels (*p* < 0.01), while the other treatment groups showed no significant differences (Figure 4D); 1 and 10 nmol/L NMB significantly increased the *Bcl-2/Bax* mRNA expression ratio (*p* < 0.01 and *p* < 0.05) (Figure 4E). Compared with the control group, treatment with 1 and 1000 nmol/L NMB significantly increased *Caspase-3* mRNA expression levels (*p* < 0.01), while treatment with other concentrations did not result in any significant differences in *Caspase-3* mRNA expression levels (Figure 4F).

Protein analysis results showed that (Figure 4G), compared with the control group, NMB treatment significantly inhibited Bax protein expression (*p* < 0.01) (Figure 4H). Treatment with 1 and 10 nmol/L NMB significantly increased Bcl-2 protein expression (*p* < 0.01), whereas treatment with other concentrations (0.1, 100, and 1000 nmol/L) significantly inhibited Bcl-2 protein expression levels (*p* < 0.01) (Figure 4I); NMB treatment (except 0.1 nmol/L) significantly increased the Bcl-2/Bax protein ratio (*p* < 0.01) (Figure 4J). Treatment with NMB inhibited Caspase-3 protein expression to varying degrees; specifically, treatment with 100 nmol/L NMB significantly inhibited Caspase-3 protein expression (*p* < 0.05), while the other concentrations (0.1, 1, 10 and 1000 nmol/L) all significantly inhibited Caspase-3 protein expression (*p* < 0.01) (Figure 4K). Original uncropped Western blot images corresponding to Figure 4G are provided in Supplementary Figure S3.

### 3.5. NMB Regulates Steroidogenesis in Porcine GCs

**Figure 5:**
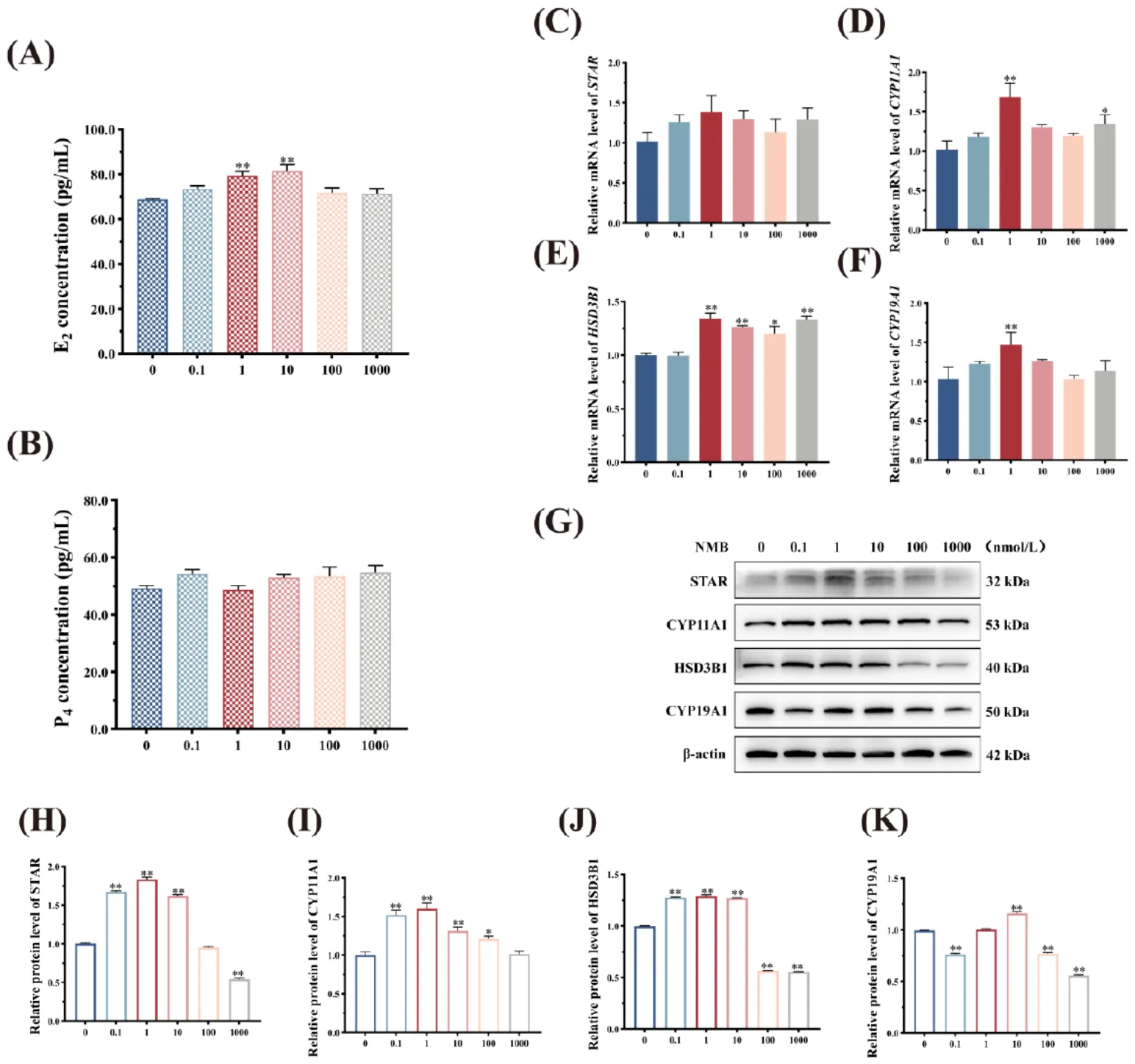
Effects of different concentrations of NMB on steroidogenesis in porcine GCs. (**A**) Levels of E_2_ in the culture medium of porcine GCs after stimulation with different concentrations of NMB. (**B**) Levels of P_4_ in the culture medium of porcine GCs after stimulation with different concentrations of NMB. (**C**) Effect of different concentrations of NMB on *STAR* mRNA expression. (**D**) Effect of different concentrations of NMB on *CYP11A1* mRNA expression. (**E**) Effect of different concentrations of NMB on *HSD3B1* mRNA expression. (**F**) Effect of different concentrations of NMB on *CYP19A1* mRNA expression. (**G**) Western blot detects the expression of STAR, CYP11A1, HSD3B1 and CYP19A1 proteins at different concentrations of NMB. (**H**) Gray analysis of STAR protein. (**I**) Gray analysis of CYP11A1 protein. (**J**) Gray analysis of HSD3B1 protein. (**K**) Gray analysis of CYP19A1 protein. Data are presented as the mean ± SEM from three independent biological replicates (n = 3), with each sample measured in duplicate within each independent experiment. Compared with the control group, * *p* < 0.05, ** *p* < 0.01. Original uncropped Western blot images corresponding to Figure 5G are provided in Supplementary Figure S4.

This experiment further investigated the effect of NMB stimulation on the ability of GCs to produce steroid hormones by measuring the secretion levels of E_2_ and P_4_ in the supernatant of cell culture medium. Compared with the control group, E_2_ secretion levels were significantly elevated (*p* < 0.01) following treatment with 1 and 10 nmol/L NMB, while other concentrations had no significant effect (Figure 5A); in contrast, treatment with different concentrations of NMB had no significant effect on P_4_ secretion levels (Figure 5B).

In this experiment, qPCR was further used to assess the effects of NMB on the expression levels of genes related to steroid hormone synthesis in porcine GCs (*STAR*, *CYP11A1*, *HSD3B1*, and *CYP19A1*). The results are shown in Figure 5C–F. Compared with the control group, NMB promoted *STAR* gene expression, but the difference was not significant (Figure 5C); 1 and 1000 nmol/L NMB significantly promoted the mRNA expression level of the *CYP11A1* gene (*p* < 0.01 and *p* < 0.05), while no obvious changes in *CYP11A1* mRNA expression levels were observed in the remaining treatment groups (Figure 5D). Treatment with 1, 10 and 1000 nmol/L NMB all significantly increased *HSD3B1* mRNA expression levels (*p* < 0.01), while treatment with 100 nmol/L NMB significantly increased its mRNA expression levels (*p* < 0.05) (Figure 5E); treatment with 1 nmol/L NMB significantly increased *CYP19A1* mRNA expression levels (*p* < 0.01), whereas mRNA expression levels did not change significantly at other concentrations (Figure 5F).

Western blot analysis was performed to detect the expression levels of proteins involved in steroid hormone synthesis in porcine GCs. The results showed that treatment with certain concentrations of NMB promoted the expression of these proteins in porcine GCs (Figure 5G). Original uncropped Western blot images corresponding to Figure 5G are provided in Supplementary Figure S4. Compared with the control group, treatment with 0.1, 1 and 10 nmol/L NMB all significantly promoted STAR protein expression (*p* < 0.01), whereas treatment with 1000 nmol/L NMB significantly inhibited STAR protein expression (*p* < 0.01) (Figure 5H). Certain concentrations of NMB (0.1, 1 and 10 nmol/L) significantly promoted CYP11A1 protein expression (*p* < 0.01), while 100 nmol/L NMB significantly promoted CYP11A1 protein expression (*p* < 0.05), whereas 100 nmol/L NMB had little effect on CYP11A1 protein expression levels (Figure 5I); 0.1, 1 and 10 nmol/L NMB significantly promoted HSD3B1 protein expression (*p* < 0.01), whereas at other concentrations of NMB (100 and 1000 nmol/L), HSD3B1 protein expression levels were significantly inhibited (*p* < 0.01) (Figure 5J). Under the influence of 10 nmol/L NMB, CYP19A1 protein expression levels increased significantly (*p* < 0.01), whereas under the influence of 1 nmol/L NMB, CYP19A1 protein expression levels showed no significant change, and CYP19A1 protein expression levels in the other treatment groups were significantly inhibited (*p* < 0.01) (Figure 5K).

### 3.6. NMB Regulates GC Proliferation and Apoptosis Through NMBR

Results from the stimulation of porcine GCs with NMB indicated that, after 24 h of treatment with NMB at certain concentrations, cell viability and altered proliferation-related markers of porcine GCs were promoted, apoptosis was inhibited, and the expression of genes and proteins related to steroidogenesis was enhanced. Considering the overall cellular responses observed across the preceding experiments, 1 nmol/L NMB for 24 h was selected as a representative effective concentration for subsequent experiments. To investigate whether the regulatory effects of NMB on porcine GCs are mediated by a specific receptor, this study combined NMB treatment with the NMBR antagonist (PD168368), using the NC group (no NMB or PD168368 treatment) as a control, to observe their effects on cell viability and steroidogenesis.

**Figure 6:**
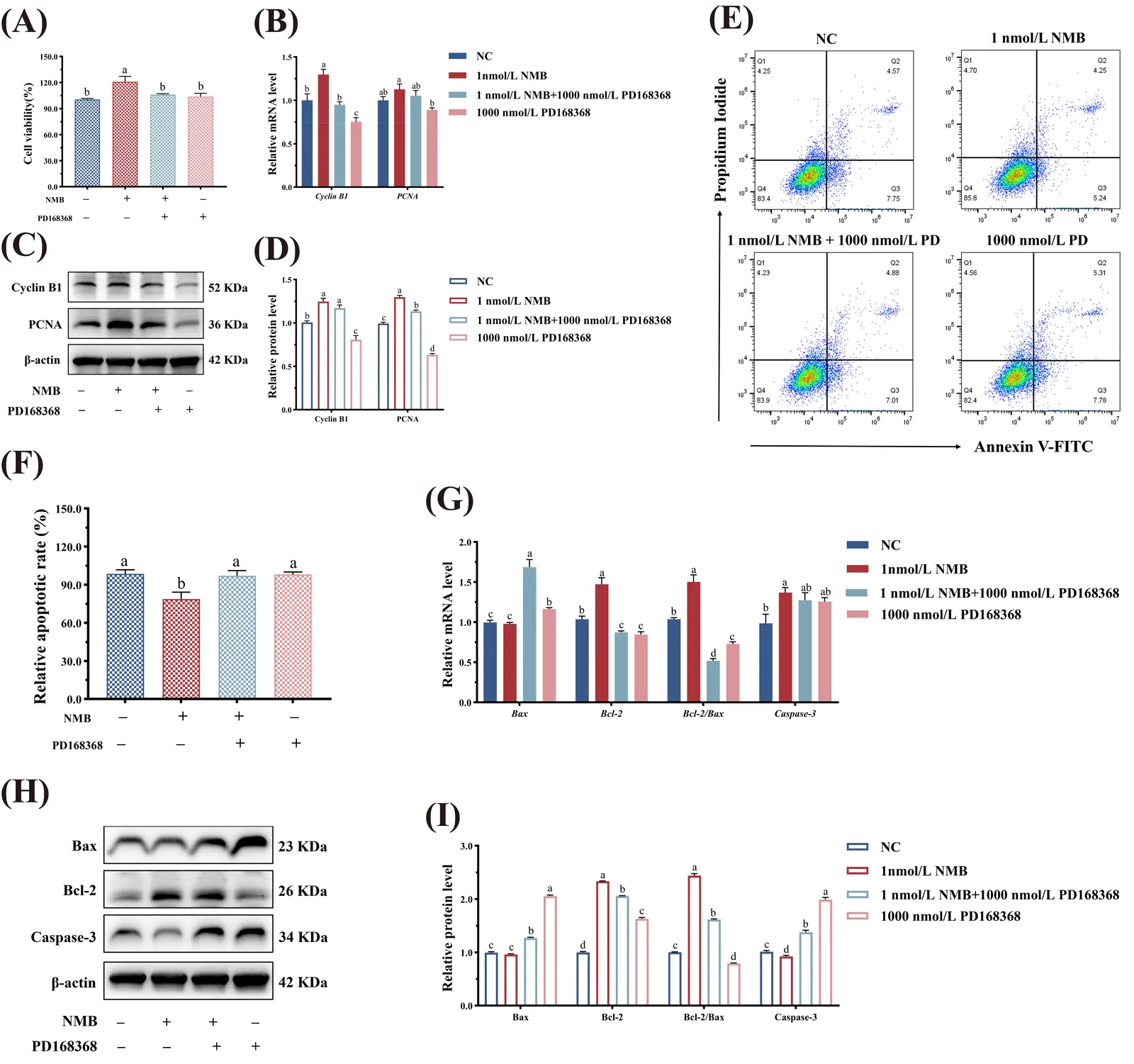
NMBR antagonist reverses the effect of NMB on cell proliferation and cell apoptosis in GCs. (**A**) The effect of PD168368 and NMB treatment on cell viability as detected by the CCK-8 assay. (**B**) The mRNA expression levels of proliferation-related genes (*Cyclin B1* and *PCNA*) in porcine GCs under PD168368 and NMB treatment. (**C**) Western blot detected the expression of Cyclin B1 and PCNA proteins under PD168368 and NMB treatment. (**D**) Results of the proteins intensity analysis in Figure 6C. (**E**) Representative results of flow cytometry assay. (**F**) The quantification of apoptotic cell populations. (**G**) The mRNA expression levels of apoptotic-related genes in porcine GCs under PD168368 and NMB treatment. (**H**) Western blot detected the expression of apoptotic-related proteins under PD168368 and NMB treatment. (**I**) Results of the proteins intensity analysis in Figure 6H. Data are presented as the mean ± SEM from three independent biological replicates (n = 3), with each sample measured in duplicate within each independent experiment. Values with different superscript letters are significantly different (*p* < 0.05). Original uncropped Western blot images corresponding to Figure 6C,H are provided in Supplementary Figure S5.

The CCK-8 assay was used to assess cell viability; as shown in Figure 6A, 1000 nmol/L PD168368 significantly inhibited NMB-induced cell proliferation. Analysis of proliferation-related genes revealed (Figure 6B) that 1000 nmol/L PD168368 significantly inhibited the promotion of *Cyclin B1* mRNA expression levels by NMB (*p* < 0.05), but had no significant inhibitory effect on NMB-induced *PCNA* expression. Analysis of proliferation-related proteins (Figure 6C,D) showed that 1000 nmol/L PD168368 significantly inhibited Cyclin B1 protein expression (*p* < 0.05), but had no significant effect on NMB’s promotion of Cyclin B1 protein expression; 1000 nmol/L PD168368 significantly inhibited the promotion of PCNA protein expression by NMB (*p* < 0.05) and significantly inhibited PCNA protein expression (*p* < 0.05). Original uncropped Western blot images corresponding to Figure 6C are provided in Supplementary Figure S5.

Flow cytometry results showed (Figure 6E,F) that 1000 nmol/L PD168368 significantly antagonized the anti-apoptotic effect of NMB on porcine GCs (*p* < 0.05). Analysis of apoptosis-related genes revealed (Figure 6G) that 1000 nmol/L PD168368 significantly increased the mRNA expression level of the pro-apoptotic gene *Bax* (*p* < 0.05) and significantly inhibited the suppressive effect of NMB on this gene (*p* < 0.05). Similarly, 1000 nmol/L PD168368 significantly suppressed the mRNA expression level of the anti-apoptotic gene *Bcl-2* (*p* < 0.05) and significantly inhibited the promoting effect of NMB on it (*p* < 0.05). Furthermore, 1000 nmol/L PD168368 significantly inhibited the *Bcl-2/Bax* ratio (*p* < 0.05) and significantly suppressed the promoting effect of NMB on this ratio (*p* < 0.05); however, 1000 nmol/L PD168368 had no significant effect on the mRNA expression level of the *Caspase-3* gene. Analysis of apoptosis-related proteins revealed (Figure 6H,I) that 1000 nmol/L PD168368 significantly promoted the expression of pro-apoptotic proteins Bax and Caspase-3 (*p* < 0.05), significantly inhibited the expression of the anti-apoptotic protein Bcl-2 (*p* < 0.05), and significantly reduced the Bcl-2/Bax ratio (*p* < 0.05). Furthermore, 1000 nmol/L PD168368 significantly enhanced the inhibitory effects of NMB on Bax and Caspase-3 (*p* < 0.05) and significantly inhibited the stimulatory effects of NMB on Bcl-2 and the Bcl-2/Bax ratio (*p* < 0.05). Original uncropped Western blot images corresponding to Figure 6H are provided in Supplementary Figure S5.

### 3.7. NMB Regulates Steroidogenesis in GCs Through NMBR

**Figure 7:**
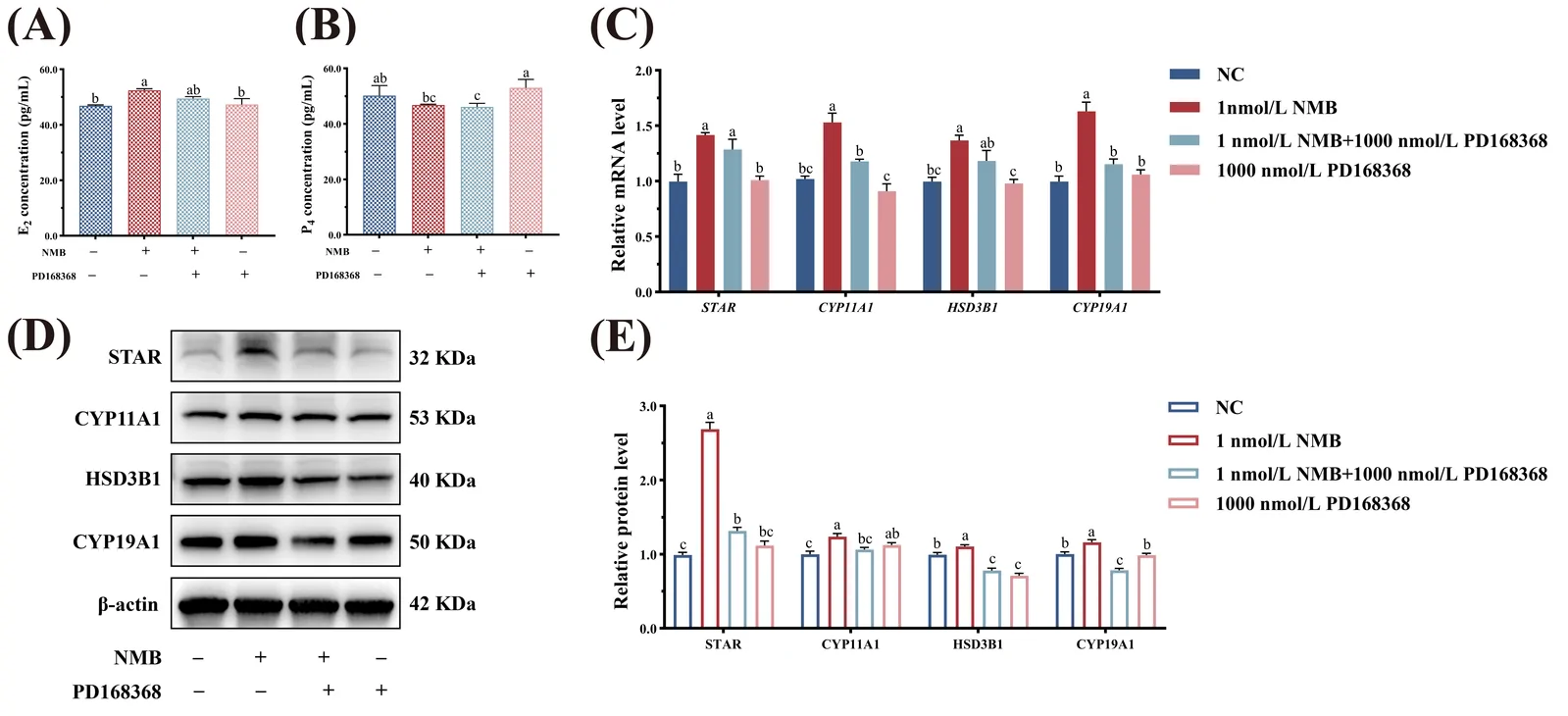
NMBR antagonist reverses the effect of NMB on steroidogenesis in GCs. (**A**) Levels of E_2_ in the culture medium of porcine GCs under PD168368 and NMB treatment. (**B**) Levels of P_4_ in the culture medium of porcine GCs under PD168368 and NMB treatment. (**C**) The mRNA expression levels of steroid hormone synthesis-related genes (*STAR*, *CYP11A1*, *HSD3B1* and *CYP19A1*) in porcine GCs under PD168368 and NMB treatment. (**D**) Western blot detected the expression of STAR, CYP11A1, HSD3B1 and CYP19A1 proteins under PD168368 and NMB treatment. (**E**) Results of the proteins intensity analysis in Figure 7D. Data are presented as the mean ± SEM from three independent biological replicates (n = 3), with each sample measured in duplicate within each independent experiment. Values with different superscript letters are significantly different (*p* < 0.05). Original uncropped Western blot images corresponding to Figure 7D are provided in Supplementary Figure S6.

Results from the analysis of E_2_ and P_4_ levels in GCs (Figure 7A,B) showed that 1 nmol/L NMB significantly increased E_2_ secretion levels (*p* < 0.05), while 1000 nmol/L PD168368 inhibited the stimulatory effect of NMB; 1000 nmol/L PD168368 significantly increased P_4_ secretion levels (*p* < 0.05). Subsequently, qPCR and Western blot analyses were performed to further investigate the effects of PD168368 on the expression of genes and proteins related to NMB-induced steroidogenesis. Gene expression analysis results (Figure 7C) showed that 1000 nmol/L PD168368 significantly inhibited the stimulatory effect of NMB on *CYP11A1* and *CYP19A1* mRNA expression levels (*p* < 0.05), whereas its antagonistic effect on NMB-induced increases in *STAR* and *HSD3B1* mRNA expression levels was not significant. Protein analysis results (Figure 7D,E) showed that 1000 nmol/L PD168368 significantly inhibited the expression levels of the steroid hormone synthesis-related proteins CYP11A1 and HSD3B1 (*p* < 0.05) and antagonized the stimulatory effect of NMB on these proteins (*p* < 0.05). Original uncropped Western blot images corresponding to Figure 7D are provided in Supplementary Figure S6.

## 4. Discussion

This study focused on porcine ovarian tissue and characterized the distribution of NMBR within the porcine ovary. NMBR expression was compared among porcine follicles of different diameters to assess expression changes associated with follicular development. This study found that NMBR is widely distributed in porcine ovarian tissue and is expressed in follicles of different stages. Its expression level increases with follicle diameter, suggesting that NMB/NMBR may be extensively involved in ovarian physiological activities and is closely related to follicular development. Furthermore, IHC results showed that NMBR is primarily distributed in the GC layer of the ovary, suggesting that GCs may be important target cells for NMB to exert its effects in the porcine ovary, and providing histological evidence that NMB regulates granulosa cell function through NMBR-mediated signaling. The results of this study are consistent with previous research detecting NMB/NMBR expression in the ovaries of mice, humans, and goats [25,29,30]. The experimental results presented in this study provide an important theoretical foundation for further investigation into the effects of NMB on GCs function in the porcine ovary.

Ovarian GCs are among the most important functional somatic cells in follicular development; they not only participate in follicular growth, provide nutritional support to the oocyte and maintain the follicular microenvironment, but also perform key physiological functions such as the synthesis and secretion of steroid hormones [31]. The proliferation, apoptosis, and differentiation status of GCs are directly related to the outcome of follicular development; therefore, establishing a stable and reliable in vitro culture model is a prerequisite for investigating local regulatory mechanisms in the ovary. In this study, we successfully isolated and cultured primary porcine ovarian GCs. Through morphological observation and FSHR purity analysis, we demonstrated that the isolated cells possessed high purity and good biological viability, consistent with the findings of Chen Huali et al. and Liang Aixin et al. [32,33] regarding the biological characteristics of porcine ovarian GCs cultured in vitro. Compared with immortalized cell lines, primary porcine GCs are better able to retain the natural biological characteristics of follicular cells, making in vitro experiments more closely resemble the in vivo environment and thus more suitable as an experimental model for studying the roles of local regulatory factors [34]. Furthermore, this study employed IHC methods to examine the distribution and expression of NMBR in porcine GCs. The results showed positive expression of NMBR in GCs, indicating that the in vitro-cultured GCs retain the receptor basis for responding to NMB stimulation. Therefore, this model can be used to further investigate the direct effects of NMB on the function of porcine ovarian GCs. FSHR is a specific marker of GCs, and its expression confirmed the identity of the cultured porcine GCs. Although FSHR expression was used to support granulosa cell identity, additional GC markers and theca cell markers such as CYP17 were not evaluated. Therefore, minor contamination by theca cells cannot be completely excluded, and more comprehensive cell-type validation should be included in future studies.

GCs proliferation is a crucial foundation for maintaining cell numbers, promoting follicle growth, and facilitating follicular cavity formation. Based on this, this study first analyzed the effects of NMB on proliferation-related responses of porcine GCs. Cell viability assays indicated that NMB significantly enhanced the cell viability of porcine GCs, suggesting that NMB exerts a distinct biological effect on GCs. Cell viability is often regarded as a key indicator reflecting cellular metabolic status, proliferative potential, and overall survival capacity [35]; however, it does not directly measure cell proliferation. Therefore, the present results suggest that NMB may affect the growth-related status of GCs rather than directly demonstrating enhanced proliferation. This study further examined the expression of proliferation-related genes and proteins and found that NMB induced significant changes in the expression levels of PCNA and Cyclin B1 to varying degrees. PCNA is an important nucleoprotein involved in DNA replication and is commonly used in cell proliferation studies as an indicator of the level of proliferative activity. Cyclin B1 is a key cyclin that regulates the G2/M phase transition of the cell cycle, and changes in its expression can, to a certain extent, reflect the progress of the cell cycle and the state of mitotic entry [36,37]. Moderate proliferation of GCs is a crucial foundation for maintaining normal follicular growth, supporting oocyte development, and forming a well-structured follicular cavity [38]. Therefore, the effects of NMB on cell viability and proliferation-related molecules suggest that NMB may influence GC growth-related functions that are potentially relevant to follicular physiology. Furthermore, the downregulation observed in the 0.1 nmol/L NMB treatment group may reflect a limited cellular response at this low concentration, whereas the 10 nmol/L NMB-treated group exhibited partially inconsistent trends in the expression of proliferation-related genes and proteins, suggesting that NMB’s regulation of proliferation-related responses may occur not only at the transcriptional level but also potentially involve post-transcriptional and post-translational regulatory processes. mRNA abundance and protein expression levels do not always correlate strictly; protein levels are also jointly regulated by multiple factors, including mRNA stability, translation efficiency, protein degradation rates, and post-translational modifications [39]. Furthermore, upon co-administration of the NMBR antagonist PD168368, the NMB-induced changes in cell viability and proliferation-related markers were attenuated, supporting the involvement of NMBR in these cellular responses rather than definitively demonstrating receptor-specific regulation. The results of this study are consistent with those reported by Zeng et al. [40] who demonstrated that the specific inhibitor PD168368 downregulates NMBR, thereby inhibiting cell proliferation and promoting apoptosis in cervical cancer cells.

GCs apoptosis is another key factor determining follicular fate. One of the hallmarks of follicular atresia is an increase in programmed GCs death; therefore, the survival-versus-death balance of granulosa cells is closely related to follicular development [38]. Bcl-2, Bax, and Caspase-3 are important molecules that regulate apoptosis [41,42]. As an anti-apoptotic protein, Bcl-2 primarily promotes cell survival by maintaining mitochondrial membrane integrity and inhibiting the release of cytochrome c; Bax is a pro-apoptotic protein that increases the permeability of the outer mitochondrial membrane and activates the mitochondrial-dependent apoptotic pathway; the Bcl-2/Bax ratio is considered a key indicator reflecting the balance between anti-apoptotic and pro-apoptotic factors; caspase-3 is a critical effector molecule in the execution phase of apoptosis, and its activation leads to DNA fragmentation, chromatin condensation, and the formation of apoptotic bodies [7,43,44]. This study found that NMB treatment enhanced the expression of the anti-apoptotic molecule Bcl-2 and suppressed the expression of the pro-apoptotic molecules Bax and Caspase-3. Furthermore, NMBR receptor antagonism attenuated the anti-apoptotic effect of NMB, supporting the involvement of NMBR in the regulation of GC survival. These findings suggest that NMB may contribute to the maintenance of GC survival, although its potential effect on follicular atresia requires further in vivo investigation. Although NMB treatment significantly upregulated the transcriptional level of Caspase-3, the simultaneous decrease in Caspase-3 protein expression indicates that changes at the mRNA and protein levels were not fully consistent. The activity of caspase proteins is regulated by various post-translational modifications (such as phosphorylation and ubiquitination), and these mechanisms can alter protein stability, activation status, or selective degradation without directly reflecting changes in transcriptional levels [45]. Therefore, post-transcriptional or post-translational regulation may represent a possible explanation for the observed discrepancy between *Caspase-3* mRNA and protein levels. However, because Caspase-3 activation and post-translational modifications were not directly examined in the present study, the underlying mechanism remains to be clarified. Combined with changes in proliferation-related indicators, these findings suggest that NMB may influence multiple aspects of GC functional status, including proliferation-related responses and programmed cell death.

The capacity for steroid hormone synthesis is a key indicator for evaluating the functional level of GCs. The results of the present study showed that NMB treatment altered steroid hormone secretion and the expression of steroidogenesis-related genes and proteins to varying extents, suggesting that NMB may modulate steroidogenesis-related functions in porcine GCs. Moreover, PD168368 attenuated several NMB-induced steroidogenic responses, supporting the involvement of NMBR. STAR mediates the transport of cholesterol to the inner mitochondrial membrane and is one of the rate-limiting steps in steroid biosynthesis, while enzymes such as CYP11A1, HSD3B1, and CYP19A1 are involved in the synthesis of pregnenolone, progesterone, and estrogen, respectively [28,46]. Changes in the expression levels of these molecules may reflect alterations in the steroidogenic capacity of GCs, but do not necessarily translate directly into corresponding changes in hormone secretion. It is therefore hypothesized that the NMB-NMBR axis not only influences proliferation-related responses and apoptosis status of GCs but may also be involved in the regulation of steroidogenesis-related genes and proteins. Notably, NMBR immunoreactivity was also detected in the theca layers. Since theca cells provide androgen substrates for estradiol synthesis, NMB/NMBR signaling in these cells may also contribute to follicular steroidogenesis, although this requires further investigation. The changes in the mRNA and protein levels of steroidogenesis-related genes were not completely consistent, which may be related to post-transcriptional regulation and differences in protein synthesis and degradation. Moreover, the increase in protein levels at low-to-moderate NMB concentrations followed by a decrease at higher concentrations suggests a possible nonlinear, concentration-dependent response. Consistent with this complexity, E_2_ secretion increased only at selected NMB concentrations, whereas P4 secretion was not significantly altered. Steroid hormone synthesis is also influenced by enzyme activity and the coordinated regulation of multiple steroidogenic factors, which may partly explain the differences between gene expression and hormone secretion. Therefore, 1 nmol/L NMB should be considered a representative effective concentration rather than the optimal concentration for all steroidogenic responses, and further dose–response studies are warranted.

**Figure 8:**
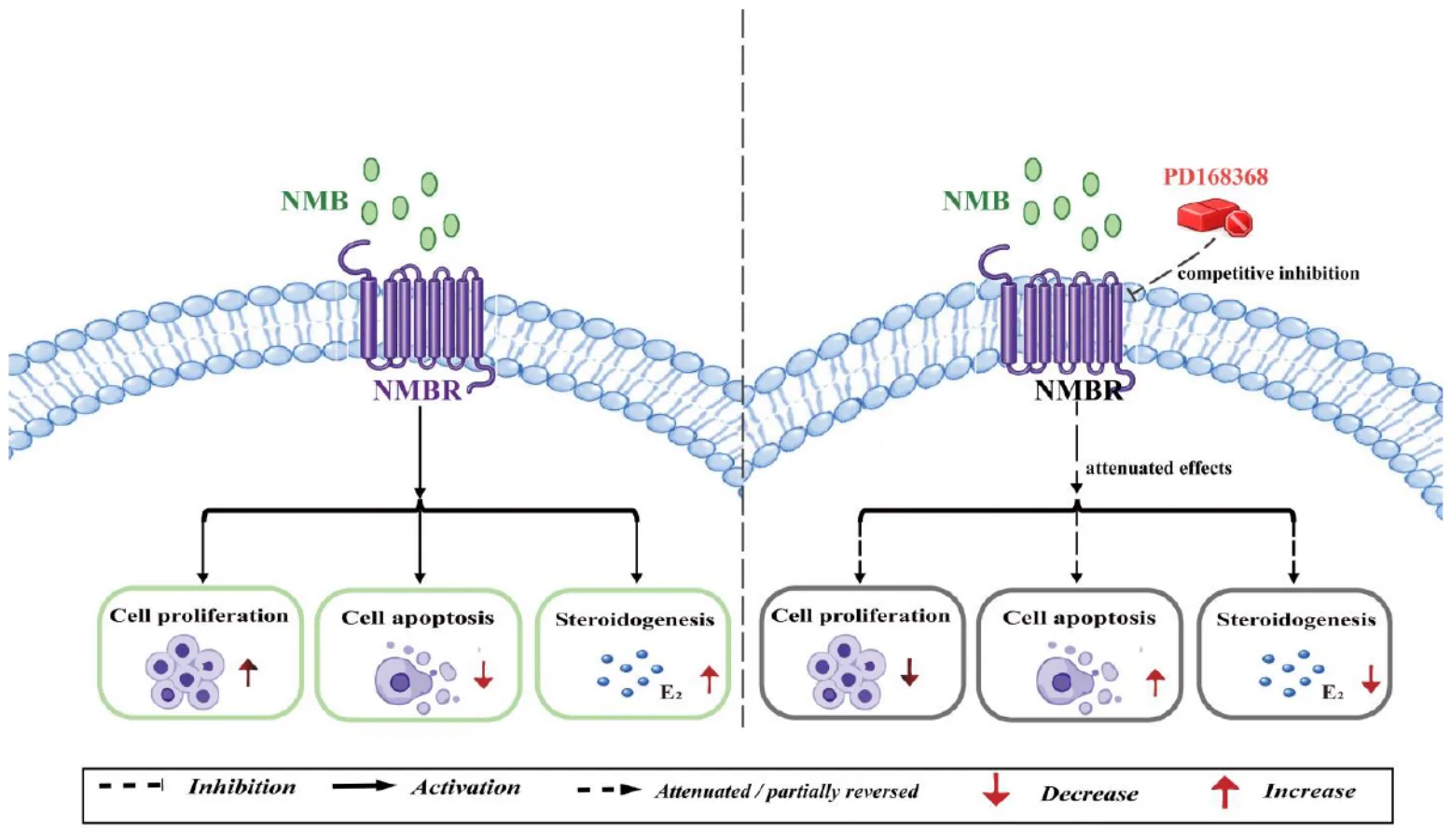
Proposed hypothetical model illustrating the potential involvement of NMB/NMBR signaling in the regulation of porcine granulosa cell function. NMB may act through NMBR to modulate GC viability, proliferation-related responses, apoptosis, and steroidogenesis. Co-treatment with an NMBR antagonist attenuated several NMB-induced cellular responses, supporting the involvement of NMBR.

In summary, the present study showed that NMBR was expressed in porcine ovaries, with NMBR being primarily localized in granulosa cells (GCs) within ovarian follicles. In addition, higher NMBR expression was observed in follicles of larger diameter. In vitro experiments further showed that NMB treatment affected GC viability, proliferation-related markers, apoptosis, and steroidogenesis, whereas the NMBR antagonist PD168368 attenuated several of these effects, supporting the involvement of NMBR in NMB-induced cellular responses. This study provides experimental evidence for further investigation of the potential role of NMB/NMBR signaling in porcine GC function and follicular physiology (Figure 8). The schematic model in Figure 8 should therefore be regarded as a hypothetical model based on the present observations rather than an established regulatory mechanism. Several limitations of the present study should be considered. Although FSHR expression was used to support the granulosa cell identity of the cultured cells, CYP17 expression or the CYP19/CYP17 ratio was not evaluated; therefore, minor contamination by theca cells cannot be completely excluded. Furthermore, proliferation was evaluated using cell viability and proliferation-related molecular markers rather than direct proliferation assays, and NMBR involvement was assessed pharmacologically without genetic validation. As NMBR is a G-protein-coupled receptor, downstream pathways involving Ca^2+^ signaling, PKC, MAPK/ERK, PI3K/AKT, or cAMP-dependent signaling may potentially contribute to the cellular responses induced by NMB. However, these downstream signaling pathways were not examined in the present study, and their involvement remains to be determined. Further studies should therefore use more stringently defined granulosa cell preparations and combine direct functional assays, genetic approaches, and signaling pathway analyses to clarify the molecular mechanisms through which NMB/NMBR signaling regulates porcine GCs function and to determine its physiological relevance to follicular development in vivo.

## 5. Conclusions

In ovarian follicles, higher NMBR mRNA and protein expression was observed in follicles of larger diameter. NMB modulated cell viability, proliferation-related markers, apoptosis, and steroidogenesis. Co-treatment with the NMBR antagonist attenuated several NMB-induced effects on GC viability, apoptosis, and steroidogenic responses, supporting the involvement of NMBR. Taken together, these findings suggest that the NMB/NMBR system may be involved in the local regulation of porcine GC function and may have a potential role in follicular physiology. Further in vivo studies are required to determine its physiological significance in porcine follicular development.

## Figures and Tables

**Table 1:** Primer sequences for qPCR.

Genes	GenBank ID	Primers Sequences (3′–5′)		Length (bp)
*β-actin*	XM_003124280.2	F: R:	CTCCATCATGAAGTGCGACGT GTGATCTCCTTCTGCATCCTGTC	114
*NMBR*	NM_001128489.1	F: R:	TAGGCCACATGATTGTCAC GACTTCCTTCCGCAACAGA	136
*STAR*	NM_213755.2	F: R:	AGTGGAACCCCAGTGTCAAG GCTCAGGCATCTCTCCAAAG	198
*CYP11A1*	NM_214427.1	F: R:	CACCCCATCTCCGTGACC GCATAGACGGCCACTTGTACC	101
*HSD3B1*	NM_001004049.1	F: R:	TTCAATCGCCACTTCGTGACC CTTCACCAGGGAGCCAACCCA	144
*CYP19A1*	XM_021083473.1	F: R:	TCCGCAATGACTTGGGCTAC GCCTTTTCGTCCAGTGGGAT	103
*PCNA*	NM_001291925.1	F: R:	CTGCAAGTGGAGAACTCGGA AGGAGAGAGTGGAGTGGCTT	161
*Cyclin B1*	NM_001170768.1	F: R:	ATTAGAGCCCCAGCCAGAGA AGCCAGATGTTTCCATGGGG	140
*BAX*	XM_013998624.2	F: R:	TAAACTGGTGCTCAAGGCCC GGCGTCCCAAAGTAGGAGAG	150
*BCL-2*	XM_021099593.1	F: R:	CTTTGAGTTCGGTGGGGTCA GGCCCATACAGCTCCACAAA	162
*Caspase-3*	NM_214131.1	F: R:	TGTGGGATTGAGACGGACAG TCCGTCCTTTGAATTTCGCC	129

The primer sequences for *CYP19A1* were adopted from a previous study by Wang et al. [28].

**Table 2:** Details of antibodies for Western blot and immunohistochemistry.

Antibodies Name	Source	Cat NO.	Dilution (WB)	Dilution (IHC)
β-actin	TransGen Biotech,	HC201	1:10,000	
NMBR	SANTA CRUZ	SC-374623	-	1:500
FSHR	Wanleibio	WLH4307	-	1:200
NMBR	Abcam	ab134141	1:1000	-
STAR	HUABIO	ER1917-23	1:500	-
CYP11A1	Biobyt	E10163	1:1000	-
HSD3B1	ABclone	A8035	1:1000	-
CYP19A11	Abcam	ab124776	1:2000	-
PCNA	SANTA CRUZ	SC-7907	1:500	-
Cyclin B1	SANTA CRUZ	SC-595	1:1000	-
BAX	Beyotime Biotechnology	AF0057	1:1000	-
BCL-2	Abcam	AB182858	1:2000	-
Caspase-3	SANTA CRUZ	SC-7148	1:1000	-
HRP-Conjugated Goat Anti-Rabbit IgG (H+L)	BOSTER	BA1054	1:10,000	-
HRP-Conjugated Goat Anti-Mouse IgG (H+L)	NCM Biotech	P8001	1:10,000	-

## Data Availability

The data that support the findings of this study are available upon request.

## Supplementary Materials

The following supporting information can be downloaded at: https://www.mdpi.com/article/10.3390/vetsci13101088/s1.

## Abbreviations

The following abbreviations are used in this manuscript:

Bax	Bcl-2 associated X protein
Bcl-2	B-cell lymphoma 2
Caspase-3	cysteine-aspartic acid protease 3
CYP11A1	cytochrome P450 family 11 subfamily A member 1
CYP19A1	cytochrome P450 family 19 subfamily A member 1
FSH	follicle-stimulating hormone
HSD3B1	hydroxysteroid dehydrogenase 3 beta type 1
LH	luteinizing hormone
PCNA	proliferating cell nuclear antigen
STAR	steroidogenic acute regulatory protein
