# Supplementary material for: Neuromedin B Regulates Cell Proliferation, Apoptosis and Steroidogenesis of Porcine Ovarian Granulosa Cells via NMBR
Source: Vet Sci. 2026 Oct 14;13(10):1088. doi: 10.3390/vetsci13101088 (PMC13709793; doi:10.3390/vetsci13101088)
Supplement: Supplementary file 1 [file vetsci-13-01088-s001.zip › Legends for Supplementary Figures S1-S6.pdf]

Figure S1 Complete uncropped Western blot images and corresponding chemiluminescence/white-light overlays showing NMBR expression in follicles of different diameters, corresponding to Figure 1C. (A, C) Complete uncropped chemiluminescence images of the membrane sections used for the detection of NMBR and β-actin, respectively. (B, D) Corresponding chemiluminescence/white-light overlays of the NMBR and β-actin membrane sections, respectively. The lanes represent follicle groups of different diameters, as indicated in the figure. Dashed boxes indicate the regions cropped for presentation in Figure 1C. MW denotes molecular weight and is expressed in kDa. NMBR and β-actin were separated on the same gel. After electrophoresis, the gel was sectioned according to their expected molecular-weight ranges, and the corresponding sections were transferred and immunoblotted separately. The expected molecular weights of NMBR and β-actin were approximately 72 and 42 kDa, respectively. The overlays in panels B and D were provided only to show the membrane-section boundaries and molecular-weight marker positions and were not used for quantification. No lanes or bands were added, removed, rearranged, or selectively modified.

Figure S2 Complete uncropped Western blot images and corresponding chemiluminescence/white-light overlays of the proliferation-related proteins (Cyclin B1 and PCNA) corresponding to Figure 3D. (A, C and E) Complete uncropped chemiluminescence images of Cyclin B1, PCNA and β-actin, respectively. (B, D and F) Corresponding chemiluminescence/white-light overlays of Cyclin B1, PCNA and β-actin, respectively. Dashed boxes indicate the regions cropped for presentation in Figure 3D. MW denotes molecular weight and is expressed in kDa. Cyclin B1 has an expected molecular weight of approximately 52 kDa, whereas the detected band migrated close to the 50-kDa molecular-weight marker. Thus, the value of 52 kDa shown in the main figure represents the expected molecular weight rather than an exact molecular weight determined from the blot. The overlay images were provided only to indicate membrane boundaries and molecular-weight marker positions and were not used for densitometric analysis. No lanes or bands were added, removed, rearranged, or selectively modified.

Figure S3 Complete uncropped Western blot images and corresponding chemiluminescence/white-light overlays of apoptosis-related proteins corresponding to Figure 4G. (A, C, E, G) Complete uncropped chemiluminescence images of the membrane sections used for the detection of Bax, BCL-2, Caspase-3, and β-actin, respectively. (B, D, F, H) Corresponding chemiluminescence/white-light overlays showing the membrane-section boundaries and molecular-weight marker positions. The overlay images were provided only to indicate membrane boundaries and molecular-weight marker positions and were not used for quantification. MW denotes molecular weight and is expressed in kDa. Dashed boxes indicate the regions cropped for presentation in Figure 4G. No lanes or bands were added, removed, rearranged, or selectively modified.

Figure S4 Complete uncropped Western blot images and corresponding chemiluminescence/white-light overlays of steroidogenesis-related proteins corresponding to Figure 5G. (A, B, D, F, H) Complete uncropped chemiluminescence images of the membrane sections used for the detection of STAR, CYP11A1, HSD3B1, CYP19A1, and β-actin, respectively. (C, E, G) Corresponding chemiluminescence/white-light overlays showing the membrane-section boundaries and molecular-weight marker positions. Major immunoreactive bands at the expected molecular weights indicated in the figure were used for densitometric analysis. The overlay images were provided only to indica CYP11A1 has an expected molecular weight of approximately 53 kDa, whereas the major immunoreactive band migrated close to the 50-kDa molecular-weight marker; therefore, 53 kDa denotes the expected rather than an experimentally determined exact molecular weight. The membrane boundaries and molecular-weight marker positions and were not used for quantification. MW denotes molecular weight and is expressed in kDa. Dashed boxes indicate the regions cropped for presentation in Figure 5G. No lanes or bands were added, removed, rearranged, or selectively modified.

Figure S5 Complete uncropped Western blot images of proteins associated with cell proliferation and apoptosis following NMB + PD169368 treatment, corresponding to Figure 6C and H. (A) Complete uncropped chemiluminescence images of the proliferation-related proteins Cyclin B1 and PCNA and their corresponding β-actin loading controls. (B) Complete uncropped chemiluminescence images of the apoptosis-related proteins Bax, BCL-2, and Caspase-3 and their corresponding β-actin loading controls. Major immunoreactive bands at the expected molecular weights indicated in the figure were used for densitometric analysis. The chemiluminescence/white-light overlays are provided only to indicate membrane boundaries and molecular-weight marker positions and were not used for quantification. Quantification was performed using the chemiluminescence images. MW denotes molecular weight and is expressed in kDa. Dashed boxes indicate the regions cropped for presentation in the corresponding main-text figure. No lanes or bands were added, removed, rearranged, or selectively modified.

Figure S6. Complete uncropped Western blot images of proteins associated with steroidogenesis following NMB + PD169368 treatment, corresponding to Figure 7D. Complete uncropped chemiluminescence images of the steroidogenesis-related proteins STAR, CYP11A1, HSD3B1, and CYP19A1 and their corresponding β-actin loading controls. Major immunoreactive bands at the expected molecular weights indicated in the figure were used for densitometric analysis. The chemiluminescence/white-light overlays are provided only to indicate membrane boundaries and molecular-weight marker positions and were not used for quantification. Quantification was performed using the chemiluminescence images. MW denotes molecular weight and is expressed in kDa. Dashed boxes indicate the regions cropped for presentation in the corresponding main-text figure. No lanes or bands were added, removed, rearranged, or selectively modified.
